# Implementation Strategy for a Mandatory Interprofessional Training Program Using an Instructional Design Model

**DOI:** 10.3390/nursrep15080274

**Published:** 2025-07-30

**Authors:** Susan Gledhill, Mary Jane McAuliffe

**Affiliations:** 1 Faculty of Health, School of Nursing, Midwifery & Paramedicine, Australian Catholic University (ACU), Banyo, QLD 4014, Australia; 2 Children’s Health Queensland Hospital and Health Service, Brisbane, QLD 4014, Australia; maryjane.mcauliffe@health.qld.gov.au

**Keywords:** ADDIE, basic life support, clinical skills, health professionals, interprofessional education, mandatory programmes, theory, nursing

## Abstract

This concept paper outlines an implementation strategy for a mandatory training programme using the ADDIE instructional design model for delivery to nurses and other health professionals in an interprofessional education (IPE) environment). **Background**: Competence in Basic Life Support (BLS) is a lifesaving requirement for health professionals in clinical settings to ensure patient safety and accreditation outcomes. It is essential that health professionals are supported in attending mandatory training, including BLS. To inform learning and teaching strategies, it is useful to apply theoretical perspectives to the development of mandatory staff training methods. However, various training models exist, and few are grounded in instructional design theory to the unique environment for BLS in IPE. **Method**: A theory-based implementation strategy is outlined for a mandatory interprofessional training programme including BLS, using the ADDIE model to enhance patient outcomes. ADDIE is an instructional design framework comprising five elements: Assess, Design, Develop, Implement and Evaluate; describing a learning methodology that can be readily applied to mandatory training in IPE. **Results**: Through its iterative capability, the ADDIE model promotes learner needs and rapid acquisition of clinical skills that improve training accessibility. The strategy can equip educators with teaching skills based on a robust theoretical model, with potential to promote nursing and health professional attendance for mandatory training. **Conclusions**: Mandatory health professional training that addresses a theory informed strategy framed by the ADDIE model can support interprofessional collaboration and consistent competency across healthcare teams. This strategy has potential to contribute by demonstrating how instructional design can be operationalised to improve the effectiveness and engaging approach to BLS training and education to the unique dynamics of an interprofessional environment.

## 1. Introduction

To optimise safe, quality healthcare, it is essential that clinicians are supported in attending mandatory training, including Basic Life Support (BLS). Competence in BLS is a vital, lifesaving requirement for all health professionals (HPs) working in clinical settings. To achieve competence in BLS, it is usually mandatory for HPs, including nursing, medical, allied health and other health workers, to regularly attend training and education. There is a strong correlation between cardiopulmonary resuscitation (CPR) and mortality, with health professional education representing the first risk mitigation strategy in terms of negative outcomes [1]. CPR is usually performed by a team in an often chaotic and always complex clinical setting, requiring collaboration, teamwork and effective communication among interdisciplinary health professionals to enhance any chance of an optimal outcome [2,3].

Moreover, there is credible support for interprofessional education (IPE) from the World Health Organisation based on years of research linking IPE to HP collaboration with successful clinical outcomes [2,3]. Recent research reports that healthcare students identify interactive, practice-based IPE as a significantly meaningful educational experience [4]. It stands to reason that delivering BSL training within a meaningful educational environment is likely to result in deeper acquisition of knowledge and skill and potentially greater retention of the skill required to sustain life in a real-world cardiac event [5].

There is evidence that for many HPs, exposure to cardiac arrest is relatively uncommon [6] while HP competence in BLS is often insufficient [7]. It is also widely known that prompt initiation of high-quality BLS can optimise outcomes and save lives [8]. Clearly, there is an imperative need for HPs to maintain competence, if not expertise, in BLS, through contextual knowledge acquisition and regular training [9]. It is also important to consider the underlying philosophy of education to inform learning and teaching strategies of mandatory training.

Philosophical viewpoints determine elements of education such as purpose, goals, values, teaching, learning and assessment strategies [10]. Philosophy underpins clinical practice requiring HPs to not only apply practical skills but also understand the context in which practical skills are applied [10,11]. This philosophical approach underpins the ADDIE framework which comprises five cyclical phases: Analyse, Design, Develop, Implement and Evaluate [12,13,14,15] which will be explained in a later section. The overall aim of using the ADDIE model is twofold: to equip educators with teaching skills based on a robust and flexible theoretical model and to enhance learning and patient outcomes in an IPE environment through improved HP attendance and upskilling during mandatory BLS and other training. Thus, the aim of this paper is to outline a theory-based implementation strategy for an interprofessional mandatory training programme, including BLS, using a well-known published instructional design framework known by its acronym, ADDIE. The training supports interprofessional collaboration and consistent competency across healthcare teams. Furthermore, the strategy contributes by demonstrating how instructional design can be implemented to improve the effectiveness and engaging approach to BLS training and education to the unique dynamics of an interprofessional environment.

This article extends existing knowledge about ADDIE by integrating instructional design strategies that have potential to be effective, efficient, engaging and specific for high-risk scenarios like BLS training. Utilising principles of IPE offers a novel approach enabling co design content that promotes role clarity and collaborative practice, team-based learning and reflective learning [3]. It supports adaptability across diverse clinical settings, relating to educational and organisational needs. This approach enhances learning and transfer of life saving skills across HPs to improve quality and safer patient outcomes [16,17].

## 2. Background

Evidence of staff competence in BLS is required to meet professional and accreditation standards in most healthcare organisations [18]. However, finding time amid a busy workload to attend mandatory training can be challenging for many HPs. Research indicates that there are several barriers to staff attendance for mandatory training including attitude, scheduling logistics and unrealistic self-perception of skill [19]. Enablers for attendance at mandatory training such as BLS have been identified as positive attitude, workplace flexibility, leadership, and redesigned, interactive training programmes [19,20]. Use of an instructional design model such as ADDIE delivered in an interprofessional environment can introduce efficiencies into a training programme that impacts patient care. In addressing the aim of this paper, a philosophical approach to implementation of the ADDIE model in a learning environment was explored.

## 3. Theoretical Foundations

This section outlines a quality learning education and training strategy for health professionals supported by a sound philosophical grounding. This is particularly important in an IPE learning environment where theory underpins broad-based clinical experience and different types and levels of disciplinary knowledge rather than training strategies that are more reliant on learning outcomes [2,21]. An evaluation of outcomes of implementation strategies explored in this paper will be reported in a later publication. It should be noted that recent research has identified that an insufficient use of conceptual frameworks in an IPE context has obfuscated evaluation results [17], hence the need to consolidate use of a conceptual framework prior to evaluation.

Education is underpinned by four main philosophies: realism and idealism (both traditional), pragmatism and existentialism (both contemporary) [10]. Both traditional and contemporary philosophies offer a good fit for implementation of mandatory HP education including BLS training.

A realism perspective is based on natural laws where humanistic knowledge is acquired through logic, abstraction and sensory perception [10]. Such knowledge is inherent in health service delivery where the human touch is a common feature in interactions between the HP and the patient. Aspects of learning within a realism approach include developing leadership which operates at a high level during cardiopulmonary resuscitation (CPR) where the HP must demonstrate leadership and authority to optimise patient outcomes.

Idealism values ideas, thoughts and mental beliefs in education [8]. In BLS training this would be providing compassionate care in valuing life in a high-risk situation. The clinical assessor facilitates the HP role in delivering safe, quality care by maintaining up-to-date mandatory training.

Compassionate care is at the heart of person-centred care and emphasises empathy, communication and patient dignity, but the core competency is compassion [22]. Idealism in healthcare refers to the belief in the intrinsic value of life and the ethical and moral obligation in healthcare. It is suggestive that there are limited direct studies on compassion in BLS training for IPE. However, related concepts such as reflective practice, ethical motivation and emotional readiness can be taught [23] and serve as an indirect pathway to facilitate compassion in BLS training. A randomised controlled study (RCT) involving first-year medical students (*n* = 287) randomised to one of two BLS training programmes [24] found that using a 15 min reflective practice after BLS training improved learners BLS skills and response time that developed empathy and self-awareness which are characteristics of compassionate behaviour. Similarly, a quasi-experimental study conducted among healthcare providers who had attended the BLS–AED course (*n* = 321) and those who had not (*n* = 421) analysed how attitudes towards repeated CPR–AED use, improved motivation, ethical responsibility and reduced professional anxiety closely linked to compassionate values [20]. Further identified were emotional and systemic barriers to effective BLS, that included fear of harm, litigation and lack of confidence suggesting that compassion can be limited by the organisation and psychological factors. These findings underline the importance of indirectly integrating compassionate care, reflective practice, idealism, and attitudinal training towards a person-centred approach in BLS training for health professionals.

The philosophical position of pragmatism is relevant to learning in healthcare settings [25]. From a pragmatist’s perspective, knowledge is viewed as evolving and experiential with a focus on critical thinking and scientific processes [10,25]. This perspective supports mandatory training where a learner-centred approach offers opportunity to apply critical thinking, scientific knowledge of the human body and practicing skill in responding to the deteriorating patient.

The existentialist view is that a learner can choose and explore their identity through engaging in activities that are emotionally meaningful [10]. Mandatory training provides an opportunity for HP participation in group and individual simulation activities with the ability to reflect and share their experience with the clinical assessor, enhancing meaningfulness of the training experience.

## 4. Instructional Theories and Design

While various training models exist, few are grounded in instructional design in the unique environment for BLS in IPE [26]. Instructional theories and design have an important part in the development of instructional materials to facilitate learning and performance. Knowledge and skill such as is required for BLS and other mandatory training outcomes, is strengthened by blending learning theories in the instructional design (ID) programme through use of theory-based strategies that support acquisition of adaptive expertise in healthcare training environments [2,27]. The theory of cognitivism, for example, recognises the importance of prior knowledge and skills that HPs bring to training in acquiring and assimilating new learnings [28]. A cognitivist perspective will allow HPs to decide at the start of a BLS assessment, for example, how much practice they require before undertaking their assessment [29].

In contrast to cognitivism, constructivist learning is founded on assimilation of the learner’s understanding and connections in an interactive social environment that contribute towards resilience in facing challenges which frequently occur in the clinical environment [28]. Mandatory training encapsulates a constructivist learning environment that motivates the HP both intrinsically and extrinsically. For instance, a constructivist approach enables the HP to practice mandatory BLS skill on high-fidelity mannequins before a final assessment [5]. Whichever approach is applied, clinical assessors should use open-ended questions to promote HP critical thinking, thus providing motivation to assimilate new learning [30].

Clinical assessors should also maintain a zone of proximal distance, known as Vygotsky’s ‘intentional scaffolding’ to facilitate and motivate learning on a developmental trajectory [10]. Scaffolding in the design of education benefits all learning preferences and improves knowledge and skills as the HP learns increasingly complex levels of practical skill [31,32].

Healthcare provides an effectively complex environment in which to deliver training and education, often requiring effective communication and teamwork across multidisciplinary staff members [33]. Enhanced teamwork may result from a greater understanding of other professional roles, responsibilities and role relationships that occur within a multidisciplinary learning environment [20]. Thus, the ADDIE model offers a framework to organise learning within a multidisciplinary environment [34]. The following section outlines the application of the ADDIE model.

## 5. Method—Mandatory Training Process Using the ADDIE Model

Integration of learning is especially important when the components of instruction are planned. The heart of instructional design is the design for the active learner. In the context of poor HP attendance at mandatory training, the authors investigated learning methods that would encourage greater HP attendance. The ADDIE model was selected as an appropriate instructional model for mandatory training in a healthcare environment due to its wide application, flexibility with goal setting and ease of use [15,35,36]. The Interprofessional Core Competencies for Interprofessional Collaborative Practice: Version 3 (2023) reflects Values and Ethics; Roles and Responsibilities; Communication; Teams and Teamwork, and underpins the implementation strategies below [3] by aligning each phase of the competencies to build BLS competence and collaborative practice [3].

### 5.1. Figure 1: Elements of the Instructional Design ‘ADDIE’ Model

As noted, the ADDIE model encompasses five cyclical phases: Analyse, Design, Develop, Implement, Evaluate [31,32]. The five-step iterative process is often used by instructional designers and is applicable to mandatory training including BLS, due to its flexibility, systematic approach and promotion of rapid skill acquisition [29]. The ADDIE process focuses on learning needs, goals, learning strategies and evaluation of relevant training skills [31]. The five cyclical phases incorporate:

### 5.2. Analysis—Best Approach to Design and Develop

- Assessing the organisations readiness by engaging key stakeholders in the process. Support and communication from Leadership is critical, ensuring alignment with the co design of the training [3].
- Consider organisational requirements around mandatory training and BLS.
- Analysing the health professional (HP) learner profiles (learner needs, cultural and special needs, working backgrounds and learning environment) and professional standards, and how mandatory practical training processes will be undertaken and the specific content to be taught.
- Analysing the broad goals of training and using task analysis (skills, knowledge, and communication) required by the HP. Discussing objectives and expectations of key stakeholders, underlying philosophies to be used and training resources and constraints.
- Analysing achievements, for example, quality of HP skill development and evaluating increased HP attendance rates.
- Determining training and competency needs for the BLS clinical assessors undertaking BLS assessments and requirements of other educational experts delivering mandatory training.
- Identifying the psychological safety of the team and HPs using BLS scenarios.

### 5.3. Design—Using the Analysis Information to Inform Design of the Learning and Assessment Resources

- Benchmarking involves comparing performances against known standards using appropriate assessment measures, that is, identifying learning outcomes and evaluating how the HP achieved successful completion of the assessment (learning objectives).
- Designing and selecting appropriate instructional strategies, for example, applying e-learning, assessment processes that are underpinned by the philosophies of idealism, realism, pragmatism and existentialism using blended learning theories of cognitivism and constructivism.
- Determining organisational requirements of the sequence of BLS skills to achieve.
- Engage the multidisciplinary stake holders in the co design to build clear communication to limit resistance.

### 5.4. Develop—Learning Materials Are Created

- Utilising instructional strategies to facilitate the learning objectives and validate the learning resources for the mandatory training sessions. Planning the logistics for the training (flexible scheduling can accommodate shift workers, venue, clinical assessors, tools, high fidelity mannequins). Shared resources can reduce duplication and promote collaboration.
- Embedding motivational learning aspects, for example, Keller’s ARCS Motivation Model incorporates Attention, Relevance, Confidence and Satisfaction components [26]. Research has shown that student motivation and interest in an IPE setting can be increased through the use of design strategies including video simulations, application of real-world scenarios, interactivity through gaming via online digital platforms that are cost effective and open-ended questions [26,38,39].
- Other design learning strategies that are associated with IPE include role play and inter-disciplinary or case-based group activities [38]. Create learning that builds compassion and communication. For the patient and self-care for the HPs.
- BLS training scenarios in an IPE setting. Rubrics for assessing interprofessional collaboration during training.
  Ensuring that training aligns with safety and quality standards.

### 5.5. Implementation—Delivering the Training to HP

- Start with a pilot group to allow for iterative refinement from initial data from HP feedback and facilitators to inform adjustments to the co design for continuous improvement and adaptation. Engage key stakeholders and HPs with the co design.
- Preparing the training setting to engage the HP for the training, for instance, provide feedback during practice, ensuring appropriate ergonomics of equipment and assessment processes.
- Providing professional development for the facilitator in IPE competencies by role modelling collaborative behaviours to manage team-based challenges, reflection, debrief and structured feedback. Delivering a combination of purposeful and interactive facilitation to the HPs [40].

The IPE implementation plan in Table 1 outlines the integration of compassion into BLS training using the ADDIE model.

### 5.6. Evaluation—Ensure Quality Training and Quality Learning Assessment Outcomes

- Confirming that training resources are accurate and up to date.
- Ensuring a safe work environment during BLS training and assessment.
- Reviewing and updating content to maintain quality.
- Empirical validation from pilot group looking at evaluation metrics of pre- and post-implementation surveys by HPs and clinical assessors of BLS competency and IPE collaboration, assessor skill level observation or use of an assessment tool. Qualitative feedback of the HPs experience by reviewing what was successful, what was learnt and what needs changing. Organisational data tracking of HPs skill competence aligning with accreditation standards to support future sustainability. Repeat ADDIE again, if required.

## 6. Evaluation

Quality and safety considerations are embedded in each stage of ADDIE to promote desired learning outcomes. The model utilises a systematic process for training development for HPs that is reliable [41,42]. Instructional design can also be instrumental in accomplishing planned changes. Evaluation of the learning setting should form part of the overall evaluation of the mandatory training and education process. This aspect of the instructional design process will be reviewed in the following sections.

### 6.1. The Learning Setting

Evaluating the setting determines the usefulness and effectiveness of mandatory training delivered within an educational environment. While numerous methods of evaluating the outcome of training and education have been described [28,39], a realist evaluative approach which is framed in terms of context (C) mechanism (M) and outcomes (O) is recommended [43,44]. In this approach, data is collected using multi methods followed by analysis of the ‘C M O’ concepts [43,44]. To provide balance, the following section outlines an alternative evaluation method that could be utilised in the training process.

### 6.2. P Model Incorporating Presage–Process–Product

Another method that can be used to evaluate the learning and teaching setting is the 3P model incorporating presage–process–product [45]. Presage is described as planning and design which incorporates context and characteristics of the learners and facilitators; process reflects content delivery; and product is the outcome of student learning, with all three Ps being analysed to evaluate the overall effectiveness of the learning and teaching environment.

### 6.3. Task Analysis

The aim of the ADDIE model is to improve attitude, knowledge and skills of the HP through [instructional] design of the mandatory training programme [39]. Task analysis of training includes (a) task; (b) knowledge; (c) skills and (d) attitudes of the HP to mandatory training [39,41]. Bloom’s Taxonomy has long been the standard and remains useful for curriculum development applicable to the ADDIE model with the following concepts of cognition (knowledge), affective (attitude) and mandatory motor skills that require higher order thinking [46] while Fink’s taxonomy offers a an alternative in developing learning objectives appropriate for BLS training by focusing on learning that is contextual with authentic real world experiences [46]. The point here is that alternative options can be explored during content development.

### 6.4. Post Implementation Evaluation

It is important that there is an evaluation of the ADDIE instructional design model post implementation of the learning and teaching programme. A useful model to follow for post implementation evaluation is Kirkpatrick’s educational outcomes framework which has been successfully utilised in both IPE and in ADDIE designed training and education programmes [2,13]. Evaluation criteria appraise four learning outcome-based domains: reaction, learning, behaviour and results that can be combined with other evaluation models to provide a broad and accurate reflection of the success of the programme [13]. The evaluation process also provides an assessment of any adjustments that need to be made to the design and mode of delivery.

## 7. Results

The literature has shown that through its iterative capability, the ADDIE model promotes rapid acquisition of clinical skills that can reduce time spent when attending mandatory BLS training. There is also an obvious link between higher order critical thinking, enhanced clinical practice among nurses and improved patient outcomes. Theoretical-based learning is enhanced when learners are provided with opportunities to connect knowledge components in structuring connectivity between information [38,39] which can be facilitated from within a learning environment. From a practical implication perspective, and as previously noted, a positive attitude towards BLS training has also been identified as an enabler for mandatory training attendance. Moreover, motivational factors can easily be incorporated into the ADDIE model to enhance motivation to participate in mandatory training in an IPE environment [26].

## 8. Discussion

There are several different ways to effectively educate and upskill HPs on BLS. A recent RCT study compared mannequin-based CPR simulation training with virtual training among a group of nursing undergraduate students randomly assigned to each group (*n* = 73) [47]. The content of both modes of education was based on the ADDIE model. The authors found that while knowledge and attitudes improved significantly among students in each group, student performance was superior among students who participated in simulation-based training. Despite technological issues associated with virtual training, the authors recommended a multimodal approach to training. The study highlights the flexibility and utility of ADDIE as an instructional design model which was accommodated across multimodal training.

The importance of basing practical training on sound theoretical principles in a healthcare environment was highlighted in an observation and control study that sought to analyse the effects of blended learning based on the ADDIE model [27]. Findings revealed an improvement in theoretical knowledge, practical skills, self-directed learning abilities, critical thinking skills and teaching satisfaction among nursing staff members [27]. In comparing conventional nurse education delivered to a control group, with blended learning based on the ADDIE model delivered to an observation group, significantly higher scores of critical thinking among the observation group were reported [27]. While noting limitations of the study including small sample size (*n* = 87), lack of long-term follow-up and potential evaluation bias, the authors concluded that teaching based on the ADDIE model could improve self-learning and theoretical knowledge that enhanced clinical nursing practice [27].

Further evidence of the utility of ADDIE as an instructional design model was found in a study that applied the ADDIE model to develop a mobile application (app) that would educate and support self-care for patients undergoing breast cancer surgery. During development of the app, the authors noted that ADDIE supported content that appealed to multiple senses [35] which accommodates different learning needs for students.

The ADDIE model has also been utilised in an Australian-based pharmacy preceptor training study [13]. Pharmacist preceptors (*n* = 28) completed an ADDIE design preceptor training programme and reported the interactivity and networking aspects as highlights of the programme. While the study was limited by the low number of preceptor training completions, the authors noted the positive response from participants who underwent the training as well as the flexibility of the model as a mode of delivery. The authors predict the model will be expanded across a broad range of interprofessional healthcare training sites going into the future [13].

A quasi-experimental study conducted among 55 HPs attending BLS–AED training revealed that repeated BLS–AED training improved attitudes associated with responding to patient deterioration that impacted positively on patient survival rates [20]. Study findings also revealed that the more highly trained HPs were, the more likely they were to have a positive attitude towards BLS–AED training compared to peers with limited experience [20]. Scheduling regular skill refreshers with immediate feedback has also been identified as a motivating factor for staff attendance [20,48,49].

While interprofessional collaborative education and training practice to improve competence is important for healthcare professionals who commonly practice in a complex team environment, there are some barriers to be considered. In acknowledging a recent trend towards collaborative health education, research reports that healthcare is still delivered in professional silos, often a consequence of traditional disciplinary-based hierarchical approaches to education [49]. A siloed approach overlooks the benefits of planned scheduling in a multidisciplinary educational environment. Organisational barriers that impede HP attendance at mandatory training in an IPE environment can be extrinsic including cost, workforce demands, staff shortages, and intrinsic such as negative organisational culture and quality of the instructor/course [19,49]. On an individual HP level, barriers to attendance include scheduling conflicts and resistance to learning in an IPE environment [19,33]. There is also the issue of limited knowledge and understanding of different healthcare roles among HPs which can result in lack of skill in collaborative teamwork [49]. Similar findings that hierarchical professional differences and negative perceptions of teamwork could act as barriers to learning have been identified [49]. A barrier to attendance and undertaking assessments may also be a factor contributing to low attendance for mandatory training. It is also clear that further research is required to identify the impact of instructional design and other methods of training and education on health outcomes [49,50]. Enablers for attendance at mandatory BLS training include staff involvement in redesigning the instructional design programme, interactivity within the training and relevance of content [19,40] which can be accommodated in the ADDIE model.

Other instructional design models include the ARCS-V (Attention, Relevance, Confidence, Satisfaction, and Volition) Motivation Model which has a strong motivational element and has been successfully integrated with the ADDIE model [26]. Lean Six Sigma (LSS) has also been successfully employed as an instructional mode of delivery of BLS training [51]. In a pre- and post-pilot study, the authors reported a reduction in total BLS training time for participants (*n* = 140) and staff (*n* = 12) after implementation of the LSS mode of delivery which also resulted in successful recertification of BLS for participants in the pilot study. Generalizability was limited due to the small numbers involved in the pilot study with the authors further noting a lack of published research into the use of LSS for mandatory training [51].

The broader range of literature confirming versatility and successful outcomes from the application of ADDIE [15,27,35,41,52] compared to other models of instructional design influenced conceptualisation of the model by the authors of this paper, who further aimed to address the lack of publications on implementation and teaching components within an IPE environment [40].

Moreover, limited research criticising the ADDIE model could be found. In a review of instructional design models, a comparison of 113 papers concluded that ADDIE was the most preferred [53]. This was further supported in later research that applied the ADDIE model to competency-based education [54]. The only negative implication of using ADDIE was in its description of being vague and a macro-instructional design model with the authors recommending that delivery of education using ADDIE be specific and contextual [12].

The literature demonstrates an ethical responsibility to promote learning for HP to support professional development in mandatory training including BLS [55]. This includes instructional learning strategies to promote inclusion of all learners. A potential limitation to the strategy includes the role of the trainer and clinical assessor facilitators undertaking BLS assessments. Using blended learning theories of cognitivism and constructivism relies on the facilitator to promote learning rather than work from expert teacher style for BLS assessments [28,30]. Effective facilitation is particularly important in IPE where the learning ultimately results in collaborative practices. In a BLS situation, for example, there can be life and death consequences if the collaborative practice is not as effective as it could be. To explore the perceptions and experiences of HP facilitators in IPE in Qatar, an exploratory case study approach was adopted by researchers [52]. Facilitator participants (*n* = 21) ranged across several health disciplines. Themes that were explicated included ‘Drivers to facilitator involvement’, ‘Student participation’, ‘Facilitator participation’, and ‘Organisational support’. Unsurprisingly, organisational support for IPE was found to be a strong factor behind positive facilitation experiences. Such experiences were underpinned by motivation and commitment to IPE. Recommendations for future research made by the authors included direct observation which could include video recording of the IPE facilitated session to generate a deeper understanding of the facilitation process and relevant issues. Other recommendations included protected time for IPE, professional development for facilitators and professional development points to motivate HP attendance and participation in IPE.

Facilitation in IPE requires an inclusive learning environment, bringing equality, diversity and right issues to the training [55,56]. It is essential for facilitators to provide a positive and equitable learning environment that values diversity and promotes equality and autonomy of the HP undertaking mandatory training including BLS [56].

## 9. Limitations and Recommendations

The authors acknowledge that the study is limited by its conceptual approach and lack of empirical data. The aim of the paper was to demonstrate how instructional design underpinned by theory can be implemented. This was considered important by the authors in extending knowledge, particularly in view of lacking publications detailing implementation of ADDIE within IPE [40]. The authors recommend further research that investigates outcomes including attendance rates, level of organisational support and competency levels of health professionals who attend mandatory training delivered using the ADDIE instructional design within IPE. Qualitative research or mixed methodology studies that explore motivation to attend mandatory training and education sessions within an IPE setting are also recommended.

## 10. Conclusions

This paper has conceptualised theoretical underpinnings and recommended processes to implement an interprofessional mandatory training programme using a well-known published instructional design framework known by its acronym, ADDIE. The overall aim is to provide educators with a facilitative theoretical approach that has potential to motivate HP attendance and maintain competence in essential practical skills such as BLS through regular training within IPE. Attendance by HPs for mandatory training is well-identified as a challenge for both the health professional and the organisation. Many organisations struggle to meet their compliance targets for HPs undertaking mandatory training and assessment and are at risk of not meeting accreditation requirements. This situation potentially places patients within the organisation at risk of BLS being performed by staff who may not be competent to deliver such vital quality care. By drawing on the current literature, this conceptual paper has shown that application of the ADDIE instructional model may provide a structured framework for interprofessional BLS training; however, its real-world impact requires future validation. Combining different approaches to health professional education underpinned by sound theory has potential to improve HP compliance in attending and undertaking mandatory training including BLS.

## Figures and Tables

**Figure 1:**
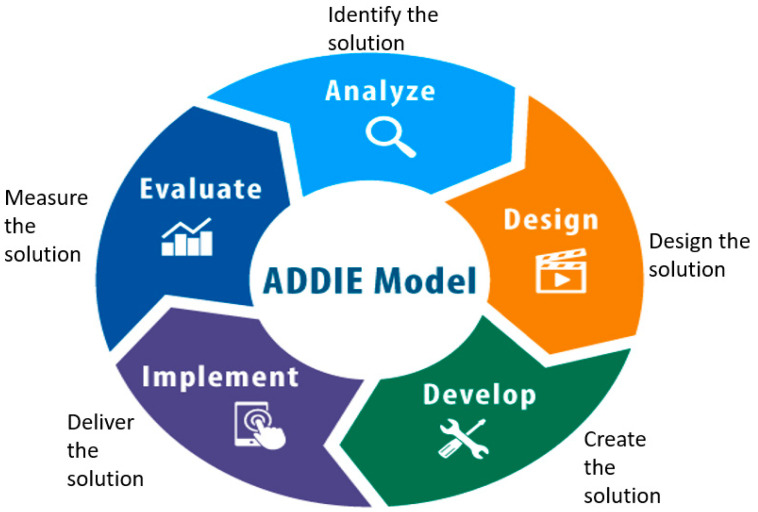
Elements of Instructional Design ADDIE Model (Source: Adapted from [37]).

**Table 1:** ADDIE Implementation Plan for BLS training in an IPE.

ADDIE Phase Applicable to BLS Training	IPE Competencies	BLS Specific Strategies	ExpectedOutcomes	Suggested Training Tools	Promote Compassion
Analysis Identify learner needs, roles and skill gaps across health profession. Broad goals for the training.	Roles/Responsibilities, Values/Ethics. Assess team dynamics of the clinical assessors from different professions, role clarity and communication barriers. Assess organisational readiness.	Identify learning needs across HPs and clinical in BLS—AED adults and children’s assessments.	Clear understanding of team’s ability and training needs.	Needs/task analysis of training. BLS assessment reviewed with IPE educational experts. Analyse organisational compliance data requirements.	Identify emotional and psychological safety of the team and HPs using BLS scenarios.
Design Create learning objectives and inclusive and collaborative content assessment.	Interprofessional communication, Teams/Teamwork. Collaborative scenarios and role-based learning.	Choose instructional design strategies (simulation mannequins with feedback). Design assessment methods.	Defined learning goals, inclusive and relevant training plans that algins with organisation.	Lesson plans, relevant scenarios and key learning objectives.	Objectives that promote empathy and person-centred care
Develop Resources, simulation, and assessments training material.	Interprofessional communication, Roles/Responsibilities. Create content and input from multiple professions.	CPR mannequins with skill guide feedback, AED trainers and skill stations. Script guides for clinical assessors.	Functioning training equipment and resources for clinical assessors.	Logistical access and scheduling for training. Instruction, guides, IPE assessments, scenarios, videos, video simulations, role play, gaming, BLS learning on digital platform. Training for HPs aligns with quality standards.	Create learning that promotes compassion and communication and support during emergencies. Self-care for HPs.
Implementation Deliver training through workshop, simulation and team practice.	Teams/Teamwork, Interprofessional communication. Facilitate interprofessional participation and clinical assessor training and rotating Team Leader role.	Real time BLS simulation, closed loop communication and feedback. Pilot group and iterative refinement.	Teamwork improved, engaged collaborative BLS training and compliance data.	Training schedule, simulation guides and equipment. Attendance tracking.	Encourage compassionate interaction by the clinical assessor and HP during simulation and feedback on empathy. Dedicated time for reflective Practice end of the session.
Evaluation Assess learning performance and feedback for improvement	Values/Ethics, Roles/Responsibilities. Use peer review, team debrief and self-assessment from HPs and clinical assessors.	Skill checklist, scenario debrief and pre/post feedback survey.	Data to inform co design and refinement. Quality and continuous improvement to enhance IPE. Repeat ADDIE if required.	Evaluation metrics: qualitative pre/post survey feedback (skill improvement for HPs and clinical assessors. Iterative refinement. reflective Practice.	Observe and discuss compassionate behaviour for the patient during emergencies. Use peer review and reflective practice from clinical assessors and HPs.

## Data Availability

No new data were created or analyzed in this study. Data was sourced from literature cited.

## Public Involvement Statement

.No public involvement in any aspect of this research.

## Guidelines and Standards Statement

This manuscript was drafted against the SQUIRE-EDU (Standards for QUality Improvement Reporting Excellence in Education): Publication Guidelines for Educational Improvement for this concept manuscript.
